# Development of polymorphic EST microsatellite markers for the sand fly, *Phlebotomus papatasi* (Diptera: Psychodidae)

**DOI:** 10.1186/s13071-018-2770-3

**Published:** 2018-03-09

**Authors:** Omar Hamarsheh, Mehmet Karakuş, Kifaya Azmi, Kaouther Jaouadi, Mohammad Reza Yaghoobi-Ershadi, Andreas Krüger, Ahmad Amro, Mohamed Amin Kenawy, Mostafa Ramadhan Dokhan, Ziad Abdeen, Mary Ann McDowell

**Affiliations:** 1 Department of Biological Sciences, Faculty of Science and Technology, Al-Quds University, Jerusalem, Palestine; 2 Department of Parasitology, Faculty of Medicine, Ege University, Izmir, Turkey; 3 Department of Biochemistry and Molecular Biology, Faculty of Medicine, Al-Quds University, Jerusalem, Palestine; 4 Department of Medical Epidemiology, Laboratory of Transmission, Control and Immunobiology of Infections (LR11IPT02) Institut Pasteur de Tunis, 13 Place Pasteur BP-74, 1002 Tunis-Belvedere, Tunisia; 5 Department of Medical Entomology & Vector Control, School of Public Health, Tehran University of Medical Sciences, Tehran, Iran; 6 Department of Tropical Medicine, Military Hospital Hamburg, Bernhard-Nocht-Straße 74, 20359 Hamburg, Germany; 7 Faculty of Pharmacy, Al-Quds University, Jerusalem, Palestine; 8 Department of Entomology, Faculty of Science, Ain Shams University, Abbassia, Cairo, 11566 Egypt; 9 Department of Zoology, Faculty of Science, University of Sabratha, Sabratha, Libya; 10 Faculty of Medicine, Al-Quds University, Jerusalem, Palestine; 11 Department of Biological Sciences, Eck Institute for Global Health, Galvin Life Science, University of Notre Dame, Notre Dame, IN 46656 USA

**Keywords:** *Phlebotomus papatasi*, *Leishmania major*, Microsatellites, Population structure

## Abstract

**Background:**

*Phlebotomus papatasi* is a widely distributed sand fly species in different tropical and sub-tropical regions including the Middle East and North Africa. It is considered an important vector that transmits *Leishmania major* parasites, the causative agents of cutaneous leishmaniasis. The development of microsatellite markers for this sand fly vector is of high interest to understand its population structure and to monitor its geographic dispersal.

**Results:**

Fourteen polymorphic microsatellite markers were developed with simple di-, tri- and tetra-nucleotide repeats. The *F* statistics calculated for the 14 markers revealed high genetic diversity; expected heterozygosity (*H*e) ranged from 0.407 to 0.767, while observed heterozygosity (*H*o) was lower and ranged from 0.083 to 0.514. The number of alleles sampled fall in the range of 9–29. Three out of 14 markers deviated from Hardy-Weinberg expectations, no significant linkage disequilibrium was detected and high values for inbreeding coefficient (*F*_IS_) were likely due to inbreeding.

**Conclusions:**

The development of these functional microsatellites enable a high resolution of *P. papatasi* populations. It opens a path for researchers to perform multi locus-based population genetic structure analyses, and comparative mapping, a part of the efforts to uncover the population dynamics of this vector, which is an important global strategy for understanding the epidemiology and control of leishmaniasis.

**Electronic supplementary material:**

The online version of this article (10.1186/s13071-018-2770-3) contains supplementary material, which is available to authorized users.

## Background

*Phlebotomus papatasi* sand flies are vectors of *Leishmania major* parasites: the causative agents of cutaneous leishmaniasis in the Middle East and North Africa. The wide geographical range and the extensive use of insecticides, climate change, wars and natural catastrophes could affect population dynamics of vectors of infectious diseases [1–6]. Like most other sand flies, *P. papatasi* has received little attention by population geneticists; molecular genetic studies on this species using various markers were documented [7–10] and no new microsatellites have been developed, except five polymorphic markers developed by our group in 2006 [11–13].

Due to high polymorphism information content and fast mutation rate, microsatellites have been used with success in the past for population analysis of various insects and sand flies including *P. papatasi* [13–21]. Like other nuclear DNA markers, microsatellites found in expressed sequence tags (ESTs) are of great value as they represent a set of functional markers. High mutation rates and simple Mendelian inheritance of these loci make them appropriate for investigations on population dynamics, breeding patterns and phylogeny [22, 23]. Although selection can be expected to be operating on a small percentage of EST markers, this drawback can be largely overcome by the use of a sufficient number of markers. On the other hand, markers proved to be under selection and non-neutral should be removed from the analysis.

Research based on EST analysis suggested that the frequency of microsatellites in some organisms is greater than was expected, had a reduced occurrence of null alleles, and had high transferability to other species [24, 25]. In this study we describe the identification of a new panel of 14 polymorphic microsatellites based on our previously mined *P. papatasi* EST simple sequence repeats [16].

## Methods

One hundred and one flies originating from 19 locations in six countries have been analyzed, including two laboratory colonies and one field population from Egypt, one laboratory colony and seven field populations from Turkey, two field populations from Tunisia, three field populations from Iran, two field populations from Afghanistan, and one laboratory colony from Cyprus. DNA was extracted from five individual flies in each population using a DNA extraction kit (Invitrogen, Carlsbad, CA, USA), following the manufacturer’s instructions. The EST primers were selected from a list of EST primers which has been mined previously by our group [16] and based on the following criteria: number of tandem repeat motifs ≥ 5, no compound motifs were used, and loci were selected from different contigs to avoid linkage disequilibrium.

The PCR reactions were carried out in a 25 μl reaction mixture containing 2.5 μl 10× PCR buffer, 0.5 μl dNTP mixture, 0.15 μl of TaKaRa *Taq*, 1.2 μl of template DNA, and 0.5 μM of each primer. For PCR amplification, DNA was denatured at 94 °C for 5 min followed by 35 cycles (94 °C for 45 s, annealing for 40 s, 72 °C for 45 s), and a final extension at 72 °C for 7 min. Polymorphisms were evaluated by separating PCR products on high resolution 3.5% MetaPhore agarose gel (Lonza, Rockland, ME, USA). For accurate sizing of the polymorphic PCR products, the forward primers were labeled with 5'- fluorescent dyes (D2-D4). The PCR products were then analyzed using the automated CEQ^TM^ 8000 sequencer (Beckman Coulter, Fullerton, CA, USA) and the fragment sizes were analyzed using its fragment analysis tool. Estimates of heterozygosity, inbreeding coefficient (*F*_IS_), and allele counts were completed using the software package FSTAT version 2.9.3.2 [26]. As null alleles can overestimate *F*_IS_ values, the Bayesian based individual inbreeding model (IIM) implemented in the program INEST 2.0 [27, 28] was used to simultaneously estimate the presence of null alleles and inbreeding coefficients. INEST was run using nfb (null alleles, in breeding coefficients, and genotyping failures) and nb (null alleles and genotyping failures) models to detect the existence of inbreeding effects in our dataset. The number of cycles (MCHC iterations) was set to 500,000 and ‘burn-in’ was 50,000. Tests for Hardy-Weinberg equilibrium and linkage disequilibrium were done using the GenAlEx package [29].

## Results and discussion

Out of 721 potential microsatellites already mined in our previous work [16], 85 primer pairs were selected and optimized. Thirty-four primer pairs successfully amplified the target sequence and generated a single band of the correct size in preliminary screening using agarose gel electrophoresis. A total of 14 microsatellite markers were found polymorphic when tested on *P. papatasi* flies from different countries (Table 1).

**Table 1:** Primer sequences and locus characteristics

Locus	Accession no.	Repeat	Fragment size (bp)	Forward (5'-3')^a^	Reverse (5'-3')	Tm (°C)^b^	Gene^c^
PPEST 2	FG10856.1	(GCA)13	141	D2-TGTCAATAGTGGCTCAATGCTC	ATTAGTCGTTTATCCTTCCCCG	60	PPAI004876-PA
PPEST 10	FG117371.1	(TC)12	236	D2-ACTGAATCTTCTGCTTTCTCCATTC	TAAGGGAAGGGGCGGAAC	60	PPAI001073-PA
PPEST 11	ES347986.1	(GA)11	162	D4-GGTGGATACTTGTGACGACTGA	CCACTCAAACTAAACTGGAAAGC	60	PPAI005664-PA
PPEST 17	FG116712.1	(TGC)9	208	D4-CTGTTCAGCAAAACGAGACG	TCCCAAGTACAAAGACGGAACT	60	PPAI008660-PA
PPEST 33	FG115100.1	(GAA)15	251	D4-ATACTCCCTCAGAACTAGCCCC	TTCGTCTTCTTCTTCTTCCTCC	60	PPAI005234-PA
PPEST 34	EY215687.1	(AAAG)5	137	D3-CACCTACAGAGATGCTGGATTG	GGGCTAAAATGTGTCTTGACTTG	62	PPAI003756-PA
PPEST 40	FG114532.1	(AG)5	322	D2-TCCCAAGGCTATTAAGTCTGGT	GGCTATCGTGCAATTTTCTTCT	62	PPAI005295-PA
PPEST43	EX474024.1	(CT)8	228	D3-AAAAGAGATTTTCGGGGAAGG	GATTGTTGAAGGAGTGAAAGGG	62	PPAI008911-PA
PPEST49	FG114549.1	(AG)10	171	D2-AAACAGCTTCAATCGCTCTGAC	CTCACTCACTCTCCCTTCGTCT	60	PPAI001715-PA
PPEST68B	ES349040.1	(CA)6	195	D3-TGATTTCACCCTTGTGTTTCC	TGTGGCAACTTATTTACATCCC	57.1	PPAI009554-PA
PPEST73	EY213549.1	(GA)7	244	D3-CCAACAATCTCCTCTCTATCGC	CCCTCAAGCTAACAAACACACA	57.1	PPAI009131-PA
PPEST75	EY203801.1	(GTT)5	237	D4-TTGTCAGGAAAGGAGTTGTTCA	GTATGCAGCTCTCCCAGAAGAT	55.5	PPAI006509-PA
PPEST77	EY204214.1	(GA)8	181	D2-AATCTCAATCTGGGCAATGTGT	ACCTTCTCTGTAAATTCCCTTGG	57.1	PPAI004565-PA
PPEST85	EY208767.1	(GA)9	244	D4-GTTGAAGGGAAATTGTGAGGAT	ATGGACATTTGTGGACTCTGC	60	PPAI003566-PA

^a^Forward primer labeled with either D2, D3, or D4 fluorescent dye at 5' end

^b^Annealing temperature

^c^Gene code based on full *P. papatasi* genome assembly (available at www.vectorbase.org

The expected heterozygosity (*He*) for all loci was relatively higher than observed heterozygosity (*H*o), ranging between 0.083–0.514 (Table 2) suggesting a heterozygote deficiency, which has been reported previously for *P. papatasi* microsatellites [11]. The gap between *H*o and *H*e values, suggests the presence of null alleles, isolation, genetic drift, population sub structuring (Wahlund effect) or inbreeding [30]. However, this gap may be due to high inbreeding as revealed by relatively positive high *F*_IS_ values calculated by FSTAT and INEST 2.0 programs.

**Table 2:** Summary of descriptive statistics of *P. papatasi* microsatellite markers

Locus	*Ho* ^a^	*He* ^b^	*F*_*IS*_^c^ *FSTAT/ INEST*	No. of alleles	HWE^d^
PPEST33	0.102	0.572	0.842/0.808	29	0.020
PPEST77	0.296	0.697	0.557/0.573	11	0.339
PPEST2	0.083	0.407	0.802/0.666	9	0.113
PPEST75	0.192	0.481	0.600/0.595	9	0.120
PPEST11	0.307	0.64	0.565/0.526	10	0.125
PPEST73	0.218	0.489	0.552/0.632	10	0.019*
PPEST68	0.415	0.767	0.464/0.466	17	0.598
PPEST85	0.182	0.570	0.657/0.640	15	0.132
PPEST10	0.315	0.648	0.513/0.536	13	0.020*
PPEST34	0.514	0.75	0.315/0.328	13	0.224
PPEST17	0.337	0.544	0.360/0.456	11	0.244
PPEST40	0.376	0.649	0.435/0.521	13	0.451
PPEST49	0.219	0.517	0.580/0.630	14	0.804
PPEST43	0.278	0.651	0.598/0.589	11	0.025*

^a^Observed heterozygosity

^b^Expected heterozygosity

^c^Wright Inbreeding coefficients (*F*_IS_), calculated using both FSTAT and in INEST 2.0

^d^*P*-values of the exact test for Hardy-Weinberg equilibrium (*P < 0.05 considered significant)

The deviance information criterion (DIC) calculated from the “nfb” model gave a lower value (23,612.759) than the “nb” model (24,696.659) supporting the inbreeding model and its strong effect (Additional file 1: Table S1 and Additional file 2: Table S2) rather than the null allele model.

One limitation of using EST-SSRs is that they generally considered less polymorphic than other microsatellite marker types, but have the benefit of an efficient and economic method and reduced occurrence of null alleles because the DNA sequences flanking SSRs from transcribed regions are relatively stable [25]. Therefore, the markers described here are very promising and can be used with confidence for population structure studies of this sand fly vector.

A few loci, markers PPEST73, PPEST10, and PPEST43, deviated significantly from Hardy-Weinberg expectations, and therefore provide caution of the utilization of these markers. None of the loci were in linkage disequilibrium (LD); all genotypic disequilibrium comparisons showed *P*-values above the 5% nominal level (0.00055). The number of alleles per locus ranged from 9 to 29 alleles, with the higher number of alleles observed in our study being likely due to the higher resolution of fluorescence-based genotyping as well as the inclusion of many field caught flies. These markers may have transferability among other species. However, tests for transferability should be completed on all sand fly species to extend the usefulness of these markers for interspecies studies.

Mining EST sequences is an effective strategy to identify functional microsatellites in *P. papatasi* sand flies. The polymorphic microsatellite markers discovered in this study will be useful for further population structure analysis, comparative mapping between populations or species, and determining the changes occurred as a result of selection.

## Conclusions

The decreased expenses of development, and lower frequency of null alleles are significant benefits of EST microsatellites, they considered valuable and appropriate markers for future population genetic studies and comparative mapping in *P. papatasi*. Transferability evaluation should be completed, in order to extend the benefits of these markers to other sand fly species.

## Additional files

Additional file 1: **Table S1.** *F*_is_ calculations using Bayesian based individual inbreeding model (IIM) implemented in the program INEST 2.0. (XLSX 11 kb)

Additional file 2: **Table S2.** The deviance information criterion (DIC) comparison results between “nb” and “nbf” models. (XLSX 35 kb)

## Abbreviations

DIC: deviance information criterion
EST: expressed sequence tags
*F*_IS_: Wright's inbreeding coefficient
*H*e: expected heterozygosity
*H*o: observed heterozygosity
HWE: Hardy-Weinberg equilibrium
LD: linkage disequilibrium
SSR: simple sequence repeat
